# Additional value of ^18^F-FDG PET/CT response evaluation in axillary nodes during neoadjuvant therapy for triple-negative and HER2-positive breast cancer

**DOI:** 10.1186/s40644-017-0117-5

**Published:** 2017-05-25

**Authors:** Mette S. van Ramshorst, Suzana C. Teixeira, Bas B. Koolen, Kenneth E. Pengel, Kenneth G. Gilhuijs, Jelle Wesseling, Sjoerd Rodenhuis, Renato A. Valdés Olmos, Emiel J. Rutgers, Wouter V. Vogel, Gabe S. Sonke, Marie-Jeanne T. Vrancken Peeters

**Affiliations:** 1 Department of Medical Oncology, Netherlands Cancer Institute, Plesmanlaan 121, 1066 CX Amsterdam, The Netherlands; 2 Department of Nuclear Medicine, Netherlands Cancer Institute, Plesmanlaan 121, 1066 CX Amsterdam, The Netherlands; 3 Department of Surgical Oncology, Netherlands Cancer Institute, Plesmanlaan 121, 1066 CX Amsterdam, The Netherlands; 4 Department of Radiology, Netherlands Cancer Institute, Plesmanlaan 121, 1066 CX Amsterdam, The Netherlands; 5 Department of Department of Radiology/Image Sciences Institute, University Medical Centre Utrecht, Heidelberglaan 100, 3584 CX Utrecht, The Netherlands; 6 Department of Pathology and Division of Molecular Pathology, Netherlands Cancer Institute, Plesmanlaan 121, 1066 CX Amsterdam, The Netherlands

**Keywords:** Breast cancer, ^18^F-FDG PET/CT, Neoadjuvant treatment, Early response monitoring

## Abstract

**Background:**

^18^F**-**FDG PET/CT can monitor metabolic activity in early breast cancer during neoadjuvant systemic therapy (NST), but it is unknown if the metabolic breast and axillary response differ. We evaluated the correlation between metabolic breast and axillary response at various time points during NST. Furthermore, we analysed if the combined metabolic response improves pathologic complete response (pCR) prediction compared to using the metabolic breast response alone.

**Methods:**

^18^F-FDG PET/CT was performed at baseline (PET1), 2–3 weeks (PET2), and 6–8 weeks (PET3) of NST in patients with triple-negative (TN) and HER2-positive node-positive breast cancer. SUVmax and ∆SUVmax were determined separately for breast and axilla. Spearman’s correlation coefficients (r) between both localisations were calculated. The accuracy of pCR total (ypT0/is,ypN0) prediction using the metabolic response in breast, axilla or both was examined using logistic regression analysis.

**Results:**

Hundred-five patients were included: 45 TN and 60 HER2-positive tumours. The metabolic response in breast and axilla correlated moderately in TN tumours (*r* = 0.57) using ∆SUVmax between PET1-PET3 and poorly in HER2-positive tumours (*r* = 0.49) using SUVmax at PET2. In TN tumours, metabolic breast response predicted pCR well without improvement after adding axillary response (c-index 0.82 versus 0.85, *p* = 0.63). In HER2-positive tumours, metabolic breast response predicted pCR poorly with improvement after adding axillary response (c-index 0.64 versus 0.72, *p* = 0.06).

**Conclusions:**

^18^F**-**FDG PET/CT response during NST differs between breast and axilla. In TN tumours, pCR total prediction can be made independent of metabolic axillary response. In HER2-positive tumours, axillary response may improve pCR total prediction. These findings may help guide PET/CT-response-based changes during NST.

**Trial registration:**

NTR NTR1797. Registered 29 May 2009, retrospectively registered.

**Electronic supplementary material:**

The online version of this article (doi:10.1186/s40644-017-0117-5) contains supplementary material, which is available to authorized users.

## Background

Neoadjuvant systemic treatment (NST) is increasingly used in early breast cancer to allow down-staging of the primary tumour to facilitate breast-conserving surgery [1]. Initially tumour-positive lymph nodes may convert into tumour-negative lymph nodes during NST which permits less aggressive treatment of the axilla as well [2]. In vivo response monitoring and adapting ineffective therapy regimens may become important additional assets of a neoadjuvant approach [3, 4].

Magnetic resonance imaging (MRI) is increasingly used as standard of care for response evaluation in the breast during NST in the Netherlands. Functional imaging with radiolabelled fluor-18-deoxyglucose (^18^F-FDG) positron emission tomography combined with computed tomography (PET/CT) can visualise the glucose metabolism in the primary tumour and affected lymph nodes. Furthermore, detection of changes in tumour glucose metabolism in response to treatment enables early response monitoring [5]. Optimal long-term outcome is seen after pathologic complete response in breast and axilla (pCR total) [6] but the sensitivity to NST may differ between both sites [7, 8]. Nevertheless, most previous neoadjuvant PET/CT studies focussed on the metabolic response of the breast alone [9–15]. Substantially fewer studies evaluated the early metabolic response of the axilla [8, 16–18], the combined response in breast and axilla [7, 9, 19, 20] or the agreement between both [21].

Therefore, the aim of our study, performed in HER2-positive and triple-negative (TN) breast cancer patients, was twofold. First, we assessed the correlation between the metabolic response in breast and axilla. Second, we evaluated the additional value of incorporating the metabolic axillary response over the breast response alone in predicting pCR total.

## Methods

We performed a prospective single-centre study with sequential PET/CT scanning before and during NST in women with primary stage II-III HER2-positive or TN breast cancer. Patients were included from September 2008 until June 2014. The institutional review board approved the study protocol and all included patients provided written informed consent. Only patients with a visible primary tumour and affected lymph nodes at baseline PET/CT were included in this analysis. Forty-five of these patients were included in a previous report [19].

### Pathological evaluation

At baseline, core biopsies were obtained from the primary tumour for pathologic diagnosis and oestrogen receptor, progesterone receptor, and HER2-status, according to Dutch national guidelines (http://www.oncoline.nl/). A marker was placed at the primary tumour site to guide surgery and pathologic evaluation. Breast conserving surgery or a mastectomy was performed based on tumour characteristics, and patient’s preference. Baseline nodal status was assessed by physical, ultrasound, and PET/CT examination with cytological evaluation by fine needle aspiration of suspicious lymph nodes. Biopsies of the primary tumour and fine needle aspiration of the lymph nodes were aimed to be obtained prior to baseline PET/CT. Patients with clinical node-negative disease underwent a sentinel node procedure (SNP) either before or after NST. In case of node-positive disease at baseline a level I-II axillary lymph node dissection was performed or the initially positive marked lymph node(s) was removed guided by marking the dominant axillary node(s) with radioactive iodine seeds (MARI-procedure) [2]. PCR was assessed by experienced breast pathologists, and was defined as no residual invasive tumour cells irrespective of in-situ lesions [6]. PCR breast, pCR axilla, and their combination (pCR total) were determined.

### Treatment

Patients with TN tumours received three cycles dose-dense doxorubicin/cyclophosphamide (AC) followed by MRI-evaluation. Patients with an unfavourable MRI response, defined as <25% reduction of the largest diameter of late enhancement, switched to three cycles capecitabine/docetaxel [CD] or three cycles carboplatin/paclitaxel [CP] [22]. Patients with a favourable response were randomized between three additional cycles of AC or CD/CP. Patients with homologous recombination deficient (HRD) tumours were randomized between three cycles CD/CP or an additional AC-cycle followed by intensified alkylating chemotherapy consisting of cyclophosphamide/thiotepa/carboplatin (CTC). Patients with HER2-positive tumours received 24 cycles weekly paclitaxel/trastuzumab/carboplatin (PTC) with trastuzumab only in weeks 7, 8, 15, 16, 23, and 24 [23]. In case of an unfavourable MRI response after 8 weeks of NST patients switched to four cycles 5-fluorouracil/epirubicin/cyclophosphamide/trastuzumab (FEC-T).

### PET/CT procedures

A PET/CT was performed at baseline (PET1), after 2 to 3 weeks of treatment (PET2), and after 6 to 8 weeks (PET3). Patients were instructed to fast for 6 hours prior to the scan and blood glucose levels were required to be <10mmol/L. Based on the patient’s body mass index 180-240MBq ^18^F-FDG was administered intravenously and 10mg diazepam was given orally to reduce ^18^F-FDG-uptake by brown fat. Following a resting period of 60 ± 10 min, in accordance with EANM procedure guidelines, a PET-scan (3.00 min per bed position and image reconstruction to 2x2x2mm voxels) of the thorax was performed according to the hanging breast protocol, using a whole-body scanner (Gemini TF; Philips, Cleveland, OH) [24]. A low-dose CT-scan (2mm slices) without intravenous contrast preceded the PET acquisition for anatomical localisation. In order to be able to make a valid comparison between scans within an individual and between individuals the same imaging system and protocol including the target time interval between ^18^F-FDG injection and PET acquisition were used throughout the study. At baseline a standard supine whole-body PET/CT was performed as well as part of disease staging.

### Image reading

The acquired PET/CT images were evaluated by a panel of experienced reviewers (BK, MvR, ST), supervised by two nuclear medicine specialists (RVO, WV). All baseline scans were qualitatively assessed for sufficient ^18^F-FDG-uptake of the primary tumour and lymph node metastases, defined as the ability to visually distinguish known tumour locations from adjacent non-malignant tissue (i.e. pathological versus physiological uptake, respectively) with an estimated ratio of >2.0, to allow subsequent quantitative response evaluation. Quantitative ^18^F-FDG-uptake of the primary tumour and the most active level I-II axillary lymph node was measured as the maximum standardised uptake value (SUVmax) within a 3D region of interest (ROI). Level III lymph nodes were not included, as these are not routinely resected during axillary clearance. If the automated ROI generation was unreliable due to a low tumour-to-background ratio, the ROI was manually drawn. In case of a complete metabolic response on the subsequent scans the baseline ROI localisation was used for calculation of the SUVmax.

### Statistical analyses

All analyses were performed separately for TN and HER2-positive tumours. Descriptive statistics were used to outline patient, tumour, and treatment characteristics. For response analyses the most active axillary lymph node was included. The absolute SUVmax values at the different time points and the relative percentage changes in SUVmax (hereafter referred to as SUVmax and ∆SUVmax respectively) were determined in breast and axilla, and their association was calculated using Spearman’s correlation coefficient (r). The association of the various PET/CT parameters at different time points with pCR was tested using logistic regression analyses and presented as the c-index (equivalent of the area under the curve [AUC] in ROC analyses). Correlation and c-index results were interpreted according to previously described classifications [25, 26]. The change in c-index when adding axillary response to a model including breast response alone was tested for significance based on the algorithm proposed by DeLong et al [27].

Data were analysed using SPSS version 22.0 (SPSS Inc. Chicago, USA) and STATA (version 13; StataCorp, College Station, TX, USA). *P*-value of <0.05 was considered statistically significant. No adjustment for multiple testing was made.

## Results

### Baseline and treatment characteristics

In total 169 patients were included. Sixteen were ineligible because of stage I disease (*n* = 5), stage IV disease (*n* = 3), missing baseline PET/CT (*n* = 4), or no trastuzumab use in case of HER2-positive disease (*n* = 4). Of the remaining 153 patients, 105 had a primary tumour and positive axillary lymph nodes, both pathologically proven and visible on PET/CT. Forty-five patients had TN and 60 HER2-positive disease (Additional file 1: Figure S1). Positive nodal status was pathologically proven in all but one patient by fine needle aspiration (Table 1). In this one patient lymph node metastases were detected by a pre-treatment SNP, however one positive axillary lymph node remained in-situ and showed ^18^F-FDG-uptake on PET/CT. Nineteen patients changed treatment after 6 to 8 weeks of therapy (i.e. after PET3). In the TN subgroup, six patients changed because of insufficient MRI response and none of them achieved a pCR breast or pCR axilla. Eleven patients switched therapy according to study protocol (ten with an HRD tumour, and one without), and one patient switched because of patient’s preference. Of these 12 patients eight achieved pCR breast and six pCR axilla and pCR total. In the HER2-positive subgroup one patient changed treatment based on an insufficient MRI response. Neither pCR breast nor pCR axilla was achieved.

**Table 1:** Baseline and treatment characteristics according to subtype

	TN		HER2+		All	
	(*n* = 45)		(*n* = 60)		(*n* = 105)	
	*n*	(%)	*n*	(%)	*n*	(%)
Age (years)						
Median (IQR)	50	(36–55)	45	(37–52)	47	(37–54)
Tumour size on MRI (mm)						
Median (IQR)	31	(22–45)	38	(22–60)	33	(22–50)
Disease stage						
II	19	(42%)	26	(43%)	45	(43%)
III	26	(58%)	34	(57%)	60	(57%)
Baseline axillary staging method						
Positive, pre-SNP^a^	1	(2%)	0	(0%)	1	(1%)
Positive, FNA	44	(98%)	60	(100%)	104	(99%)
Grade						
1–2	13	(29%)	25	(42%)	38	(36%)
3	16	(36%)	14	(23%)	30	(29%)
Unknown	16	(36%)	21	(35%)	37	(35%)
Histology						
Ductal	43	(96%)	55	(92%)	98	(93%)
Lobular	0	(0%)	4	(7%)	4	(4%)
Other	2	(4%)	1	(2%)	3	(3%)
HR-status						
ER- and PR-	45	(100%)	29	(48%)	74	(71%)
ER+ and/or PR+	0	(0%)	31	(52%)	31	(30%)
Treatment						
AC^b^	45	(100%)	0	(0%)	45	(43%)
PTC^c^	0	(0%)	60	(100%)	60	(57%)
PET assessment						
PET1 performed	45	(100%)	60	(100%)	105	(100%)
PET2 performed	35	(78%)	45	(75%)	80	(76%)
PET3 performed	38	(84%)	47	(78%)	84	(80%)

*TN* triple-negative, *HER2+* HER2-positive, *n* number of patients, *PA* pathology, *SNP* sentinel node procedure, *FNA* fine needle aspiration, *ER* oestrogen receptor, *PR* progesterone receptor, *AC* doxorubicin/cyclophosphamide, *PTC* paclitaxel/trastuzumab/carboplatin

^a^SNP performed before PET1, but remaining positive axillary lymph node in situ outside surgical region

^b^Nineteen patients switched treatment after PET3: six to capecitabine/docetaxel, ten to high-dose carboplatin/thiotepa/cyclophosphamide, three to paclitaxel (+/- carboplatin)

^c^Two patients received paclitaxel/trastuzumab/carboplatin plus pertuzumab, and one patients switched to 5-fluorouracil/epirubicin/cyclophosphamide plus trastuzumab after PET3

### Surgery and pathologic response

With the exception of one patient with progressive disease during chemotherapy who refused further treatment, all patients underwent surgery. This patient was classified as having no pCR. Thus, 104 patients underwent breast surgery: 66 breast conserving surgery and 38 a mastectomy. Pathologic axillary lymph node response was assessed by axillary lymph node dissection in 89, MARI-procedure in 13, and post-treatment SNP in two patients.

In TN tumours pCR breast was achieved in 53% (24/45), pCR axilla in 47% (21/45), and pCR total in 40% (18/45). In the HER2-positive subgroup the rate of pCR breast was 65% (39/60), pCR axilla 75% (45/60), and pCR total 57% (34/60). In total 25 patients had a discrepant pathologic response of the breast and axilla: 11 pCR breast/no pCR axilla, and 14 pCR axilla/no pCR breast.

### Triple-negative disease

Baseline PET/CT was performed in all 45 patients with TN disease, PET2 in 35, and PET3 in 38. Thirty-two patients underwent three PET/CT-scans. The median time between last chemotherapy and PET2 was 13 days (interquartile range [IQR] 13-14), and between last chemotherapy and PET3 7 days (IQR 7-8). The median SUVmax and ∆SUVmax at the different time points are summarized in Table 2, including correlation coefficients between metabolic response in breast and axilla. The best correlation between metabolic response in breast and axilla was found with ∆SUVmax between PET1-PET3, and although all patients showed a decrease in ∆SUVmax in both locations at PET3 the correlation was moderate (*r* = 0.57) (Additional file 2: Figure S2a).

**Table 2:** Correlation coefficients between the metabolic response in breast and axilla with different SUVmax variables according to subtype

	TN (*n* = 45)			HER2+ (*n* = 60)		
	median	(IQR)	r	median	(IQR)	r
SUVmax PET1						
Breast	10.7	(6.5 – 16.5)	0.42	6.8	(4.7 – 9.3)	0.38
Axilla	8.0	(4.9 – 13.8)		5.3	(3.3 – 7.6)	
SUVmax PET2						
Breast	7.9	(5.1 – 10.0)	0.36	2.8	(2.2 – 3.6)	0.49
Axilla	4.2	(3.1 – 7.2)		2.1	(1.7 – 2.5)	
SUVmax PET3						
Breast	3.5	(2.5 – 5.0)	0.33	2.0	(1.5 – 2.4)	0.14
Axilla	2.1	(1.3 – 3.6)		1.7	(1.3 – 2.4)	
ΔSUVmax (%) PET1-PET2						
Breast	-32%	(-49 – -16)	0.49	-56%	(-68 – -47)	0.30
Axilla	-33%	(-58 – -13)		-56%	(-70 – -38)	
ΔSUVmax (%) PET1-PET3						
Breast	-67%	(-77 – -49)	0.57	-69%	(-78 – -52)	0.27
Axilla	-70%	(-84 – -48)		-66%	(-79 – -50)	

*TN* triple-negative, *HER2+* HER2-positive, *n* number of patients, *IQR* interquartile range, *r* Spearman’s correlation coefficient

PCR breast prediction was most accurate using ∆SUVmax breast between PET1-PET3 (c-index 0.85) (Additional file 3: Table S1). Likewise, ∆SUVmax axilla between PET1-PET3 was best for pCR axilla prediction (c-index 0.82). The metabolic breast response, using ∆SUVmax between PET1-PET3, was well predictive for pCR total and the addition of metabolic response in the axilla using ∆SUVmax between PET1-PET3 did not further improve pCR total prediction (c-index 0.82 versus 0.85, *p* = 0.63) (Table 3).

**Table 3:** C-indices (95% confidence interval) for the prediction of pathologic complete response by metabolic response in TN and HER2-positive breast cancer

	Pathologic complete response		
	Breast	Axilla	Total
TN: ΔSUVmax PET1-PET3			
Breast	0.85 (0.72 – 0.98)	0.83 (0.69 – 0.98)	0.82 (0.66 – 0.98)
Axilla	0.82 (0.68 – 0.95)	0.82 (0.68 – 0.97)	0.83 (0.67 – 0.98)
Breast + axilla	0.86 (0.74 – 0.98)	0.86 (0.72 – 0.99)	0.85 (0.69 – 1.00)
*p*-value^*^	0.78	0.60	0.63
HER2-positive: SUVmax PET2			
Breast	0.62 (0.44 – 0.81)	0.65 (0.47 – 0.84)	0.64 (0.47 – 0.81)
Axilla	0.68 (0.52 – 0.84)	0.77 (0.62 – 0.92)	0.67 (0.51 - 0.83)
Breast + axilla	0.72 (0.56 – 0.89)	0.78 (0.63 – 0.92)	0.72 (0.57 – 0.88)
*p*-value^*^	0.11	0.06	0.06

^*^ *p*-value for the improvement in c-index by the addition of metabolic response in the axilla

### HER2-positive disease

Baseline PET/CT was performed in all 60 patients with HER2-positive disease, PET2 in 45, and PET3 in 47. Forty patients underwent three PET/CT-scans. The median time between last chemotherapy and PET2 was 6 days (IQR 5-7), and between last chemotherapy and PET3 12 days (IQR 8-14). The best correlation between metabolic response in breast and axilla was found with SUVmax at PET2, although poor (*r* = 0.49) (Additional file 2: Figure S2b). In addition, an inverse response in terms of an increase in SUVmax in one location and a decrease or no difference in the other was observed in four patients at time of PET2.

The metabolic response in the breast poorly discriminates patients who will achieve a pCR breast from patients who will not. The difference in SUVmax (∆SUVmax) in the breast between PET1-PET2 had the best discriminating performance of all PET-parameters assessed (c-index 0.64), although absolute SUVmax in the breast at PET2 showed an almost similar performance (c-index 0.62) (Additional file 4: Table S2). In the axilla, SUVmax at PET2 had the best discriminating performance to predict pCR axilla (c-index 0.77). Prediction of total pCR by SUVmax in the breast at PET2 was poor but improved to fair, although not statistically significant, when both the metabolic breast and axillary response using SUVmax at PET2 were included (c-index 0.64 versus 0.72, *p* = 0.06) (Table 3).

## Discussion

This study shows that the correlation between ^18^F-FDG PET/CT responses during NST in breast and axillary lymph nodes is moderate in triple-negative and poor in HER2-positive breast cancer. In TN disease, PET/CT response can be used to predict pCR and the breast response alone suffices to predict pCR total. Conversely, in HER2-positive disease, the accuracy of PET/CT to predict pCR is limited, while incorporating the metabolic response of both the breast and axilla may improve pCR total prediction.

Lymph node involvement at baseline and after NST is an important prognostic factor in non-metastatic breast cancer [28, 29]. Furthermore, pCR defined as no invasive tumour cells in breast and axilla is best related to long-term outcome [6]. Despite this knowledge, many previous PET/CT studies evaluated the metabolic response of the breast alone to predict pCR total, without examining if the metabolic response of the primary tumour and lymph nodes is the same [4, 11–15]. Adding information about the metabolic response of axilla may aid to predict pCR total. Studies, that did evaluate the metabolic response in breast and axilla, used different strategies to combine response information of both locations to predict pCR total. Some evaluated the response of the baseline lesion with highest FDG-uptake alone [9, 30, 31] and others used ∆SUVmax between the lesion with the highest FDG-uptake at baseline and at the subsequent scan [32, 33]. However, information may be missed if the response differs between both sites or may result in comparing a breast lesion with an axillary lymph node or vice versa if the lesion with the highest FDG-uptake changes during treatment. Dalus et al. found different SUVmax measurements for breast and lymph nodes, possibly reflecting a different biological behaviour in these two sites which may relate to selection of a sub-clone of tumour cells that spreads to the lymph nodes. Therefore, they proposed to evaluate the response of the primary tumour and axilla separately [21]. We agree with this proposal until a valid combined variable has been established. Only a few studies have described the metabolic response in breast and axilla separately and its respective association with pCR breast and pCR axilla within the same cohort [7, 34]. These studies did not evaluate the correlation between the metabolic response in both locations. Therefore our study is unique and provides important new insights for PET/CT interpretation.

We found a moderate correlation between the metabolic breast and axillary response in TN breast cancer (*r* = 0.57) without significant improvement in pCR total prediction with adding the metabolic axillary response to the breast response alone. This suggests that chemotherapy sensitivity in breast and axilla corresponds well. Therefore, the metabolic breast response alone suffices to guide NST decisions. In accordance with this, Groheux et al. did not find a better prediction of pCR total in TN disease if the axillary response was incorporated in addition to the breast response [9, 31]. Koolen et al. previously described a part of our study population and found the strongest association between the combined metabolic breast and axillary response and pCR total with an AUC of 0.93 versus 0.87 for breast response alone [19]. The statistical significance of this improvement was not tested. With the inclusion of additional patients in the current analysis, the association between the combined metabolic response and pCR total was somewhat weaker, although still good with a non-significant improvement using the combination over the breast alone (c-index 0.85 versus 0.82, *p* = 0.63) [19].

In HER2-positive breast cancer the metabolic responses in breast and axilla correlate poorly (*r* = 0.49). The ability to predict pCR breast, and pCR total by the metabolic breast response was poor (c-index 0.62, and 0.64, respectively). The addition of metabolic response in the axilla improved the pCR total prediction compared to the use of breast response alone, which was statistically near-significant (c-index 0.64 versus 0.72, *p* = 0.06). Lack of statistical significance despite a relatively large increase in c-index, might be attributable to the small sample size, and larger studies are needed to determine the added value of including the metabolic response in both locations for pCR total prediction in this subtype. In line with our results, Groheux and colleagues found an improvement in pCR total prediction in node-positive patients if the axillary response was included [30]. These and our findings suggest that if PET/CT is used for response monitoring in HER2-positive breast cancer, it should evaluate both breast and axilla, and we recommend separate evaluation of both sites rather than an unconfirmed combined parameter as described above. The use of targeted therapy in HER2-positive tumours may explain why the different response according to tumour location was more pronounced in this subtype, as it may differentially affect sub-clones with varying HER2-expression. Also, we cannot exclude that in selected cases non-specific ^18^F-FDG uptake related to regional inflammatory processes or tissue sampling may have contaminated the pathological uptake. Although we recognize this as a limitation of our study the impact on our results will be limited, especially after FNA. Furthermore, non-specific ^18^F-FDG uptake is likely to have affected both subtypes equally. Lastly, with the relatively small sample size we cannot exclude that the poor and moderate correlation of metabolic responses between locations is due to chance rather than a biological finding. However, despite only four inverse responses in the HER2-positive subtype, in relative terms, this constitutes 9% of HER2-positive cases with a PET2. Additionally, the poor correlation between metabolic and axillary response despite a decrease in both locations seems relevant as it may have implications for defining metabolic responders with different thresholds for different localizations.

In accordance with the literature we found that the best prognostic PET/CT response parameter for both pCR breast and pCR axilla is ∆SUVmax between baseline PET/CT and PET/CT after 6 weeks in TN tumours and the absolute SUVmax value at PET/CT after 3 weeks of therapy in HER2-positive tumours [9, 12, 30, 31, 35].

Our data reinforce that it is important to describe results according to breast cancer subtype due to different tumour behaviour. Subgroup analysis based on hormone receptor status within the HER2-positive cohort would have been valuable, but was not feasible due to the limited number of patients.

The inclusion of patients with sufficiently high baseline FDG-uptake for response evaluation, may have led to selection of relatively aggressive tumour types and an associated higher response rate reflecting the high pCR rate in our study. Nevertheless, sufficient baseline activity is required for PET/CT-evaluation and thus this selection reflects daily practice. Furthermore, a substantial number of patients with TN tumours switched therapy, and PET/CT-scans were only performed during the initially applied regimen. However, switches based on insufficient MRI response are assumed to have had little impact on our results as all these patients remained a pathological non-responder despite the change in treatment and it is unlikely that they would have achieved total pCR if they had continued their initially applied regimen.

Clear definitions of responders and non-responders will aid the clinical use of PET/CT during neoadjuvant breast cancer treatment. The optimal cut-off value depends on several factors as described by others including treatment regimen, timing of evaluation, breast cancer subtype, and mainly depends on the purpose of the response evaluation: identifying non-responders to change ineffective treatment or identifying responders to reduce overtreatment [35].

Several PET-parameters exist but no superiority of one over the other has been established so far. This study started in 2008 and we used the region with the highest metabolic activity (i.e. SUVmax) instead of the entire metabolically active tumour volume which has been introduced more recently. However, SUVmax has important benefits as it is convenient to use and has good reproducibility [9, 32].

PET/CT for response evaluation during NST in breast cancer is not the current standard of care and probably awaits a direct comparison with other imaging modalities. In the current study we focused on the use of PET/CT only and how to optimally use this to predict pCR total. Therefore, we cannot make a statement about the relative value of PET/CT compared to other imaging modalities, but this has been described by others [36, 37]. Nowadays, trastuzumab-labelled PET/CT scans are available with visualisation of HER2-positive lesions. This modality may improve selection of patients for anti-HER2 treatment, but its role in monitoring response is undetermined [38]. Furthermore, trials to confirm the benefit of PET/CT-response-based treatment adaptations in terms of outcome are needed [3, 4].

## Conclusion

Our study demonstrates that the correlation between metabolic response in the breast and axilla is moderate in TN and poor in HER2-positive breast cancer. Furthermore, ^18^F-FDG PET/CT can be used to evaluate the response to neoadjuvant chemotherapy in TN disease. The metabolic breast response alone, using ∆SUVmax between PET/CT at baseline and after 6 weeks treatment, predicts pCR total well and adding metabolic axillary response has no additional value. In HER2-positive tumours, pCR total prediction by the metabolic breast response alone, using SUVmax at PET/CT after 3 weeks treatment, is poor. This may be improved by evaluating both the primary tumour and axillary lymph node metabolic response in this subtype, and separate evaluation is recommended.

## Additional files

Additional file 1: Figure S1. CONSORT diagram. (PDF 65 kb)

Additional file 2: Figure S2. Correlation between the metabolic response in breast and axilla in (a) triple-negative tumours (*n* = 38; ΔSUVmax PET1-PET3) and (b) HER2-positive tumours (*n* = 45; SUVmax PET2). (PDF 178 kb)

Additional file 3: Table S1. SUVmax variables according to pCR breast and pCR axilla and their prognostic value in triple-negative breast cancer. (PDF 126 kb)

Additional file 4: Table S2. SUVmax variables according to pCR breast and pCR axilla and their prognostic value in HER2-positive breast cancer. (PDF 128 kb)

## Abbreviations

^18^F-FDG: Fluor-18-deoxyglucose
AC: Doxorubicine/cyclophosphamide
AUC: Area under the curve
CD: Capecitabine/docetaxel
CP: Carboplatin/paclitaxel
CTC: Cyclophosphamide/thiotepa/carboplatin
FEC-T: Fluorouracil/epirubicin/cyclophosphamide/trastuzumab
HER2: Human epidermal growth factor receptor-2
HRD: Homologous recombination deficient
MARI: Marking the axilla with radioactive iodine seeds
MRI: Magnetic resonance imaging
NST: Neoadjuvant systemic treatment
pCR: Pathologic complete response
PET/CT: Positron emission tomography combined with computed tomography
PTC: Paclitaxel/trastuzumab/carboplatin
ROI: Region of interest
SNP: Sentinel node procedure
SUVmax: Maximum standardised uptake value
TN: Triple-negative

## References

[1] Mieog JS, van der Hage JA, van de Velde CJ. Preoperative chemotherapy for women with operable breast cancer. Cochrane Database Syst Rev. 2007;(2):CD005002. 10.1002/14651858.CD005002.pub2 PMC7388837 17443564

[2] Donker M, Straver ME, Wesseling J, Loo CE, Schot M, Drukker CA (2015). Marking axillary lymph nodes with radioactive iodine seeds for axillary staging after neoadjuvant systemic treatment in breast cancer patients: the MARI procedure. Ann Surg.

[3] von Minckwitz G, Blohmer JU, Costa SD, Denkert C, Eidtmann H, Eiermann W (2013). Response-guided neoadjuvant chemotherapy for breast cancer. J Clin Oncol.

[4] Coudert B, Pierga JY, Mouret-Reynier MA, Kerrou K, Ferrero JM, Petit T (2014). Use of [(18)F]-FDG PET to predict response to neoadjuvant trastuzumab and docetaxel in patients with HER2-positive breast cancer, and addition of bevacizumab to neoadjuvant trastuzumab and docetaxel in [(18)F]-FDG PET-predicted non-responders (AVATAXHER): an open-label, randomised phase 2 trial. Lancet Oncol.

[5] Avril S, Muzic RF, Plecha D, Traughber BJ, Vinayak S, Avril N (2016). 18F-FDG PET/CT for Monitoring of Treatment Response in Breast Cance. J Nucl Med.

[6] Cortazar P, Zhang L, Untch M, Mehta K, Costantino JP, Wolmark N (2014). Pathological complete response and long-term clinical benefit in breast cancer: the CTNeoBC pooled analysis. Lancet.

[7] Garcia Vicente AM, Amo-Salas M, Relea Calatayud F, Munoz Sanchez MD, Pena Pardo FJ, Jimenez Londono GA (2016). Prognostic Role of Early and End-of-Neoadjuvant Treatment 18F-FDG PET/CT in Patients With Breast Cancer. Clin Nucl Med.

[8] Rousseau C, Devillers A, Campone M, Campion L, Ferrer L, Sagan C (2011). FDG PET evaluation of early axillary lymph node response to neoadjuvant chemotherapy in stage II and III breast cancer patients. Eur J Nucl Med Mol Imaging.

[9] Groheux D, Majdoub M, Sanna A, de Cremoux P, Hindie E, Giacchetti S (2015). Early Metabolic Response to Neoadjuvant Treatment: FDG PET/CT Criteria according to Breast Cancer Subtype. Radiology.

[10] Groheux D, Hindie E, Giacchetti S, Delord M, Hamy AS, de Roquancourt A (2012). Triple-negative breast cancer: early assessment with 18F-FDG PET/CT during neoadjuvant chemotherapy identifies patients who are unlikely to achieve a pathologic complete response and are at a high risk of early relapse. J Nucl Med.

[11] Humbert O, Berriolo-Riedinger A, Riedinger JM, Coudert B, Arnould L, Cochet A (2012). Changes in 18F-FDG tumor metabolism after a first course of neoadjuvant chemotherapy in breast cancer: influence of tumor subtypes. Ann Oncol.

[12] Humbert O, Cochet A, Riedinger JM, Berriolo-Riedinger A, Arnould L, Coudert B (2014). HER2-positive breast cancer: (1)(8)F-FDG PET for early prediction of response to trastuzumab plus taxane-based neoadjuvant chemotherapy. Eur J Nucl Med Mol Imaging.

[13] Humbert O, Riedinger JM, Charon-Barra C, Berriolo-Riedinger A, Desmoulins I, Lorgis V (2015). Identification of biomarkers including 18FDG-PET/CT for early prediction of response to neoadjuvant chemotherapy in Triple Negative Breast Cancer. Clin Cancer Res.

[14] Pahk K, Kim S, Choe JG (2015). Early prediction of pathological complete response in luminal B type neoadjuvant chemotherapy-treated breast cancer patients: comparison between interim 18F-FDG PET/CT and MRI. Nucl Med Commun.

[15] Lee HW, Lee HM, Choi S-E, Yoo H, Ahn SG, Lee M-K (2016). The Prognostic Impact of Early Change in Standardized Uptake Value of 18F-fluorodeoxy-glucose Positron Emission Tomography after Neoadjuvant Chemotherapy in Locally Advanced Breast Cancer Patients. J Nucl Med..

[16] Straver ME, Aukema TS, Olmos RA, Rutgers EJ, Gilhuijs KG, Schot ME (2010). Feasibility of FDG PET/CT to monitor the response of axillary lymph node metastases to neoadjuvant chemotherapy in breast cancer patients. Eur J Nucl Med Mol Imaging.

[17] Koolen BB, Valdes Olmos RA, Wesseling J, Vogel WV, Vincent AD, Gilhuijs KG (2013). Early assessment of axillary response with (1)(8)F-FDG PET/CT during neoadjuvant chemotherapy in stage II-III breast cancer: implications for surgical management of the axilla. Ann Surg Oncol.

[18] Garcia Vicente AM, Soriano Castrejon A, Leon Martin A, Relea Calatayud F, Munoz Sanchez Mdel M, Cruz Mora MA (2014). Early and delayed prediction of axillary lymph node neoadjuvant response by (18)F-FDG PET/CT in patients with locally advanced breast cancer. Eur J Nucl Med Mol Imaging.

[19] Koolen BB, Pengel KE, Wesseling J, Vogel WV, Vrancken Peeters MJ, Vincent AD (2014). Sequential (18)F-FDG PET/CT for early prediction of complete pathological response in breast and axilla during neoadjuvant chemotherapy. Eur J Nucl Med Mol Imaging.

[20] Garcia Garcia-Esquinas MA, Arrazola Garcia J, Garcia-Saenz JA, Furio-Bacete V, Fuentes Ferrer ME, Ortega Candil A (2014). Predictive value of PET-CT for pathological response in stages II and III breast cancer patients following neoadjuvant chemotherapy with docetaxel. Rev Esp Med Nucl Imagen Mol.

[21] Dalus K, Rendl G, Rettenbacher L, Pirich C (2010). FDG PET/CT for monitoring response to neoadjuvant chemotherapy in breast cancer patients. Eur J Nucl Med Mol Imaging.

[22] Loo CE, Teertstra HJ, Rodenhuis S, van de Vijver MJ, Hannemann J, Muller SH (2008). Dynamic contrast-enhanced MRI for prediction of breast cancer response to neoadjuvant chemotherapy: initial results. AJR Am J Roentgenol.

[23] Sonke GS, Mandjes IA, Holtkamp MJ, Schot M, van Werkhoven E, Wesseling J (2013). Paclitaxel, carboplatin, and trastuzumab in a neo-adjuvant regimen for HER2-positive breast cancer. Breast J.

[24] Boellaard R, O'Doherty MJ, Weber WA, Mottaghy FM, Lonsdale MN, Stroobants SG (2010). FDG PET and PET/CT: EANM procedure guidelines for tumour PET imaging: version 1.0. Eur J Nucl Med Mol Imaging.

[25] El Khouli RH, Macura KJ, Barker PB, Habba MR, Jacobs MA, Bluemke DA (2009). Relationship of temporal resolution to diagnostic performance for dynamic contrast enhanced MRI of the breast. J Magn Reson Imaging.

[26] Hinkle DE, Wiersma W, Jurs SG (2003). Applied Statistics for the Behavioral Sciences.

[27] DeLong ER, DeLong DM, Clarke-Pearson DL (1988). Comparing the areas under two or more correlated receiver operating characteristic curves: a nonparametric approach. Biometrics.

[28] Donegan WL (1997). Tumor-related prognostic factors for breast cancer. CA Cancer J Clin.

[29] Mougalian SS, Hernandez M, Lei X, Lynch S, Kuerer HM, Symmans WF (2016). Ten-Year Outcomes of Patients With Breast Cancer With Cytologically Confirmed Axillary Lymph Node Metastases and Pathologic Complete Response After Primary Systemic Chemotherapy. JAMA Oncol.

[30] Groheux D, Giacchetti S, Hatt M, Marty M, Vercellino L, de Roquancourt A (2013). HER2-overexpressing breast cancer: FDG uptake after two cycles of chemotherapy predicts the outcome of neoadjuvant treatment. Br J Cancer.

[31] Groheux D, Hindie E, Giacchetti S, Hamy AS, Berger F, Merlet P (2014). Early assessment with 18F-fluorodeoxyglucose positron emission tomography/computed tomography can help predict the outcome of neoadjuvant chemotherapy in triple negative breast cancer. Eur J Cancer.

[32] Wahl RL, Jacene H, Kasamon Y, Lodge MA (2009). From RECIST to PERCIST: Evolving Considerations for PET response criteria in solid tumors. J Nucl Med.

[33] JH O, Lodge MA, Wahl RL (2016). Practical PERCIST: A Simplified Guide to PET Response Criteria in Solid Tumors 1.0. Radiology.

[34] Jung SY, Kim SK, Nam BH, Min SY, Lee SJ, Park C (2010). Prognostic Impact of [18F] FDG-PET in operable breast cancer treated with neoadjuvant chemotherapy. Ann Surg Oncol.

[35] Groheux D, Mankoff D, Espie M, Hindie E (2016). F-FDG PET/CT in the early prediction of pathological response in aggressive subtypes of breast cancer: review of the literature and recommendations for use in clinical trials. Eur J Nucl Med Mol Imaging.

[36] Liu Q, Wang C, Li P, Liu J, Huang G, Song S (2016). The Role of (18)F-FDG PET/CT and MRI in Assessing Pathological Complete Response to Neoadjuvant Chemotherapy in Patients with Breast Cancer: A Systematic Review and Meta-Analysis. Biomed Res Int.

[37] Hieken TJ, Boughey JC, Jones KN, Shah SS, Glazebrook KN (2013). Imaging response and residual metastatic axillary lymph node disease after neoadjuvant chemotherapy for primary breast cancer. Ann Surg Oncol.

[38] Gebhart G, Lamberts LE, Wimana Z, Garcia C, Emonts P, Ameye L (2016). Molecular imaging as a tool to investigate heterogeneity of advanced HER2-positive breast cancer and to predict patient outcome under trastuzumab emtansine (T-DM1): the ZEPHIR trial. Ann Oncol.

