# Supplementary figures and images for: Additional value of 18F-FDG PET/CT response evaluation in axillary nodes during neoadjuvant therapy for triple-negative and HER2-positive breast cancer
Source: Cancer Imaging. 2017 May 25;17:15. doi: 10.1186/s40644-017-0117-5 (PMC5445462; doi:10.1186/s40644-017-0117-5)

Additional file 1: Figure S1. CONSORT diagram

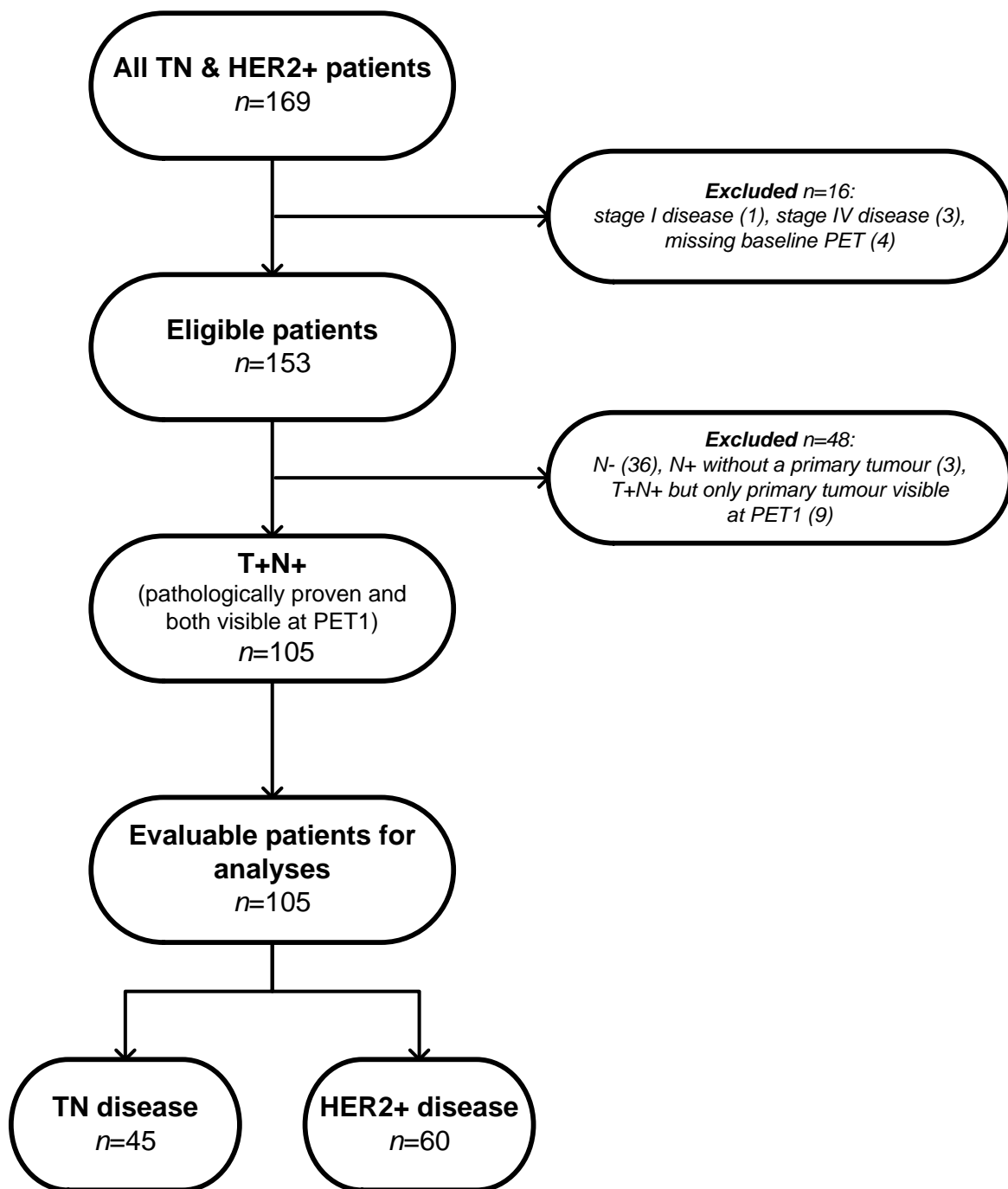

Supplement: Supplementary file 1 — CONSORT diagram. (PDF 65 kb) [file 40644_2017_117_MOESM1_ESM.pdf]
