# Supplementary material for: Additional value of 18F-FDG PET/CT response evaluation in axillary nodes during neoadjuvant therapy for triple-negative and HER2-positive breast cancer
Source: Cancer Imaging. 2017 May 25;17:15. doi: 10.1186/s40644-017-0117-5 (PMC5445462; doi:10.1186/s40644-017-0117-5)
Supplement: Supplementary file 2 — Correlation between the metabolic response in breast and axilla in (a) triple-negative tumours (n = 38; ΔSUVmax PET1-PET3) and (b) HER2-positive tumours (n = 45; SUVmax PET2). (PDF 178 kb) [file 40644_2017_117_MOESM2_ESM.pdf]

Additional file 2: Figure S2. Correlation between the metabolic response in breast and axilla in (a) triple-negative tumours (*n*=38; ΔSUVmax PET1-PET3) and (b) HER2-positive tumours (*n*=45; SUVmax PET2)

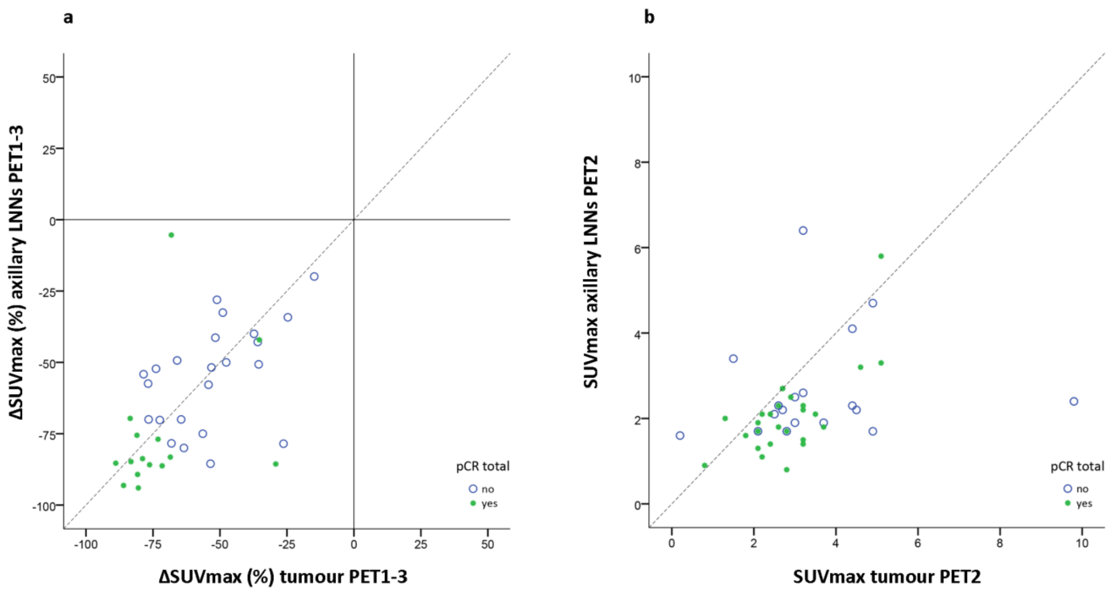

LNNs, lymph nodes; pCR total, pathologic complete response in breast and axilla
