# Supplementary material for: Additional value of 18F-FDG PET/CT response evaluation in axillary nodes during neoadjuvant therapy for triple-negative and HER2-positive breast cancer
Source: Cancer Imaging. 2017 May 25;17:15. doi: 10.1186/s40644-017-0117-5 (PMC5445462; doi:10.1186/s40644-017-0117-5)
Supplement: Supplementary file 3 — SUVmax variables according to pCR breast and pCR axilla and their prognostic value in triple-negative breast cancer. (PDF 126 kb) [file 40644_2017_117_MOESM3_ESM.pdf]

Additional file 3: Table S1. SUVmax variables according to pCR breast and pCR axilla and their prognostic value in triple-negative breast cancer

	**no pCR breast**			**pCR breast**			
	n	**median**	**(IQR)**	***n***	**median**	**(IQR)**	**c-index (95%CI)**
**Breast**							
**SUVmax PET1**	21	9.3	(7.6 - 15.7)	24	11.4	(7.3 - 17.8)	0.44 (0.27 - 0.62)
**SUVmax PET2**	17	6.9	(5.1 - 14.2)	18	8.0	(4.7 - 8.8)	0.57 (0.37 - 0.76)
**SUVmax PET3**	18	4.2	(3.4 - 10.0)	20	3.3	(1.9 - 4.3)	0.77 (0.62 - 0.92)
**ΔSUVmax PET1-PET2**	17	-25%	(-45 - -9)	18	-38%	(-50 - -19)	0.63 (0.44 - 0.82)
**ΔSUVmax PET1-PET3**	18	-51%	(-64 - -36)	20	-76%	(-81 - -68)	0.85 (0.72 - 0.98)
	**no pCR axilla**			**pCR axilla**			
	n	**median**	**(IQR)**	***n***	**median**	**(IQR)**	**c-index (95%CI)**
**Axillary LNNs**							
**SUVmax PET1**	24	7.5	(4.5 - 11.8)	21	8.0	(5.2 - 16.7)	0.43 (0.26 - 0.60)
**SUVmax PET2**	19	6.0	(3.4 - 11.7)	16	3.5	(2.7 - 5.4)	0.72 (0.56 - 0.89)
**SUVmax PET3**	21	2.8	(1.8 - 6.1)	17	1.6	(1.1 - 2.2)	0.74 (0.58 - 0.90)
**ΔSUVmax PET1-PET2**	19	-20%	(-39 - -12)	16	-57%	(-66 - -33)	0.81 (0.66 - 0.96)
**ΔSUVmax PET1-PET3**	21	-52%	(-70 - -41)	17	-84%	(-86 - -73)	0.82 (0.68 - 0.97)

pCR, pathologic complete response; n, number of patients; IQR, interquartile range; 95%CI, 95% confidence interval; LNNs, lymph nodes
