# Supplementary material for: Additional value of 18F-FDG PET/CT response evaluation in axillary nodes during neoadjuvant therapy for triple-negative and HER2-positive breast cancer
Source: Cancer Imaging. 2017 May 25;17:15. doi: 10.1186/s40644-017-0117-5 (PMC5445462; doi:10.1186/s40644-017-0117-5)
Supplement: Supplementary file 4 — SUVmax variables according to pCR breast and pCR axilla and their prognostic value in HER2-positive breast cancer. (PDF 128 kb) [file 40644_2017_117_MOESM4_ESM.pdf]

Additional file 4: Table S2. SUVmax variables according to pCR breast and pCR axilla and their prognostic value in HER2-positive breast cancer

	no pCR breast			pCR breast			
	n	**median**	**(IQR)**	*n*	**median**	**(IQR)**	**c-index (95%CI)**
**Breast**							
**SUVmax PET1**	21	7.5	(5.1 - 9.9)	39	6.6	(4.4 - 8.8)	0.58 (0.43 - 0.74)
**SUVmax PET2**	15	3.2	(2.5 - 4.5)	30	2.7	(2.2 - 3.2)	0.62 (0.44 - 0.81)
**SUVmax PET3**	15	2.1	(1.4 - 2.9)	32	2.0	(1.5 - 2.3)	0.60 (0.41 - 0.79)
**ΔSUVmax PET1-PET2**	15	-52%	(-61 - -9)	30	-64%	(-68 - -49)	0.64 (0.47 - 0.82)
**ΔSUVmax PET1-PET3**	15	-68%	(-78 - -42)	32	-70%	(-80 - -52)	0.57 (0.40 - 0.75)
	**no pCR axilla**			**pCR axilla**			
	n	**median**	**(IQR)**	*n*	**median**	**(IQR)**	**c-index (95%CI)**
**Axillary LNNs**							
**SUVmax PET1**	15	6.2	(2.8 - 7.6)	45	5.2	(2.7 - 7.6)	0.51 (0.33 - 0.69)
**SUVmax PET2**	10	2.4	(2.2 - 3.6)	35	1.9	(1.6 - 2.3)	0.77 (0.62 - 0.92)
**SUVmax PET3**	10	1.9	(1.0 - 2.8)	37	1.7	(1.3 - 2.4)	0.51 (0.27 - 0.75)
**ΔSUVmax PET1-PET2**	10	-44%	(-60 - -13)	35	-57%	(-74 - -39)	0.65 (0.47 - 0.84)
**ΔSUVmax PET1-PET3**	10	-59%	(-85 - -52)	37	-67%	(-77 - -46)	0.45 (0.23 - 0.67)

pCR, pathologic complete response; n, number of patients; IQR, interquartile range; 95%CI, 95% confidence interval; LNNs, lymph nodes
